# Supplementary material for: Machine Learning Accelerates Crystallization for Structure Determination
Source: Angew Chem Int Ed Engl. 2026 May 4;65(25):e1218503. doi: 10.1002/anie.1218503 (PMC13266958; doi:10.1002/anie.1218503)

checkCIF/PLATON report

Structure factors have been supplied for datablock(s) lcz002

THIS REPORT IS FOR GUIDANCE ONLY. IF USED AS PART OF A REVIEW PROCEDURE FOR PUBLICATION, IT SHOULD NOT REPLACE THE EXPERTISE OF AN EXPERIENCED CRYSTALLOGRAPHIC REFEREE.

No syntax errors found. CIF dictionary Interpreting this report

Datablock: lcz002

Bond precision:	C-C = 0.0096 A		Wavelength=1.54184
Cell:	a=11.6037(2)	b=13.3185(1)	c=35.1044(3)
	alpha=90	beta=91.673(1)	gamma=90
Temperature:	100 K		

	Calculated	Reported
Volume	5422.86(11)	5422.86(11)
Space group	P 21/c	P 1 21/c 1
Hall group	-P 2ybc	-P 2ybc
Moiety formula	C23 H11 Ag3 F18 N6 O4, C15 H3 Ag3 F18 N6	2(C15 H3 Ag3 F18 N6), C8 H8 O4
Sum formula	C38 H14 Ag6 F36 N12 O4	C38 H14 Ag6 F36 N12 O4
Mr	2033.83	2033.83
Dx,g cm-3	2.491	2.491
Z	4	4
Mu (mm-1)	18.724	18.724
F000	3856.0	3856.0
F000’	3875.77	
h,k,lmax	14,16,44	14,16,44
Nref	11666	11193
Tmin,Tmax	0.023,0.088	0.662,1.000
Tmin’	0.004	

Correction method= # Reported T Limits: Tmin=0.662 Tmax=1.000
AbsCorr = MULTI-SCAN

Data completeness= 0.959 Theta(max)= 78.536

R(reflections)= 0.0504(9050) wR2(reflections)= 0.1435(11193)

S = 1.099 Npar= 867

The following ALERTS were generated. Each ALERT has the format
test-name_ALERT_alert-type_alert-level.
Click on the hyperlinks for more details of the test.

Alert level C

```
PLAT042_ALERT_1_C Calc. and Reported MoietyFormula Strings  Differ     Please Check
              Calc: C23 H11 Ag3 F18 N6 O4, C15 H3 Ag3 F18 N6
              Rep.: 2(C15 H3 Ag3 F18 N6), C8 H8 O4
PLAT214_ALERT_2_C Atom F020      (Anion/Solvent) ADP max/min Ratio        4.4 prolat
PLAT214_ALERT_2_C Atom F02K      (Anion/Solvent) ADP max/min Ratio        4.2 prolat
PLAT214_ALERT_2_C Atom F02L      (Anion/Solvent) ADP max/min Ratio        4.4 prolat
PLAT221_ALERT_2_C Solv./Anion  Resd 2  F   Ueq(max)/Ueq(min) Range        4.2 Ratio
PLAT234_ALERT_4_C Large Hirshfeld Difference F020     --C02D     .       0.16 Ang.
PLAT250_ALERT_2_C Large U3/U1 Ratio for <U(i,j)> Tensor(Resd    2)        2.4 Note
PLAT342_ALERT_3_C Low Bond Precision on  C-C Bonds ..............     0.00957 Ang.
PLAT906_ALERT_3_C Large K Value in the Analysis of Variance ......      2.361 Check
PLAT911_ALERT_3_C Missing FCF Refl Between Thmin & STh/L=    0.600         36 Report
               13  0  0,    0  8  0,    0  8  1,  -6  0  2,    0  8  2,    0  8  3,
                7  0  4,    0  8  4,    2  3  6, -13  4  6, -13  4  7, -13  4  8,
              -13  4  9,   -1  7  9,    6  2 10, -13  4 10,  -1  7 10,  -4  5 11,
               -4  4 13,   11  4 15, -11  3 16,  11  4 17,   5  0 18,  -3  2 19,
                5  0 20,  -10  8 20, -10  8 21, -12  0 22,   5  0 22, -11  0 24,
                   (    6 More Missing: see the .ckf listing file)
PLAT971_ALERT_2_C Check Calcd Resid. Dens.  1.27Ang From F01F           1.55 eA-3
```

Alert level G

```
PLAT083_ALERT_2_G SHELXL Second Parameter in WGHT  Unusually Large      20.47 Why ?
PLAT142_ALERT_4_G s.u. on b - Axis Small or Missing .............    0.00010 Ang.
PLAT143_ALERT_4_G s.u. on c - Axis Small or Missing .............    0.00030 Ang.
PLAT242_ALERT_2_G Low    'MainMol' Ueq as Compared to Neighbors of       C01T Check
PLAT242_ALERT_2_G Low    'MainMol' Ueq as Compared to Neighbors of       C026 Check
PLAT242_ALERT_2_G Low    'MainMol' Ueq as Compared to Neighbors of       C027 Check
PLAT242_ALERT_2_G Low    'MainMol' Ueq as Compared to Neighbors of       C02B Check
PLAT242_ALERT_2_G Low    'MainMol' Ueq as Compared to Neighbors of       C02H Check
PLAT242_ALERT_2_G Low    'MainMol' Ueq as Compared to Neighbors of       C02O Check
PLAT242_ALERT_2_G Low    'MainMol' Ueq as Compared to Neighbors of       C029 Check
PLAT242_ALERT_2_G Low    'MainMol' Ueq as Compared to Neighbors of       C02D Check
PLAT242_ALERT_2_G Low    'MainMol' Ueq as Compared to Neighbors of       C02F Check
PLAT242_ALERT_2_G Low    'MainMol' Ueq as Compared to Neighbors of       C02G Check
PLAT242_ALERT_2_G Low    'MainMol' Ueq as Compared to Neighbors of       C02I Check
PLAT242_ALERT_2_G Low    'MainMol' Ueq as Compared to Neighbors of       C02N Check
PLAT333_ALERT_2_G Large Aver C6-Ring C-C Dist C01I     -C02E     .       1.44 Ang.
PLAT335_ALERT_2_G Check Large C6 Ring C-C Range C01I     -C02E           0.18 Ang.
PLAT434_ALERT_2_G Short Inter HL..HL Contact F00B     ..F01B     .       2.79 Ang.
                                      1-x,-1/2+y,1/2-z  =     2_645 Check
PLAT434_ALERT_2_G Short Inter HL..HL Contact F00E     ..F01D     .       2.72 Ang.
```

```
                                                       x,y,z  =      1_555 Check
PLAT434_ALERT_2_G Short Inter HL..HL Contact F00I     ..F00Z     .       2.69 Ang.
                                             -x,-1/2+y,1/2-z  =      2_545 Check
PLAT434_ALERT_2_G Short Inter HL..HL Contact F00Z     ..F01H     .       2.81 Ang.
                                             -x,-1/2+y,1/2-z  =      2_545 Check
PLAT720_ALERT_4_G Number of Unusual/Non-Standard Labels ..........        110 Note
              Ag01    Ag02    Ag03    Ag04    Ag05    Ag06    F007    F008
              F009    F00A    F00B    F00C    F00D    F00E    F00F    F00G
              F00H    F00I    F00J    F00K    O00L    F00M    F00N    F00O
              O00P    N00Q    N00R    O00S    F00T    N00U    F00V    N00W
              N00X    N00Y    F00Z    O010    F011    N012    N013    F014
              F015    N016    N017    N018    F019    N01A    F01B    F01C
              F01D    F01E    F01F    F01G    F01H    C01I    C01J    C01K
              H01K    C01L    C01M    H01M    C01N    C01O    C01P    H01P
              C01Q    C01R    H01R    F01S    C01T    C01U    C01V    H01V
              C01W    C01X    C01Y    C01Z    H01Z    F020    C021    C022
              H022    F023    C024    C025    H02A    H02B    H02C    C026
              C027    C028    C029    C02A    C02B    C02C    H02D    H02E
              H02F    C02D    C02E    H02G    C02F    C02G    C02H    C02I
              C02J    F02K    F02L    C02M    C02N    C02O
PLAT883_ALERT_1_G Absent Datum for _atom_sites_solution_primary ..     Please Do !
PLAT912_ALERT_4_G Missing # of FCF Reflections Above STh/L=  0.600        437 Note
PLAT941_ALERT_3_G Average HKL Measurement Multiplicity ..........        2.8 Low
PLAT969_ALERT_5_G The 'Henn et al.' R-Factor-gap value ..........      2.614 Note
              Predicted wR2: Based on SigI**2  5.49 or SHELX Weight 13.06
PLAT978_ALERT_2_G Number C-C Bonds with Positive Residual Density.          0 Info
```

```
   0 ALERT level A = Most likely a serious problem - resolve or explain
   0 ALERT level B = A potentially serious problem, consider carefully
  11 ALERT level C = Check. Ensure it is not caused by an omission or oversight
  27 ALERT level G = General information/check it is not something unexpected

   2 ALERT type 1 CIF construction/syntax error, inconsistent or missing data
  26 ALERT type 2 Indicator that the structure model may be wrong or deficient
   4 ALERT type 3 Indicator that the structure quality may be low
   5 ALERT type 4 Improvement, methodology, query or suggestion
   1 ALERT type 5 Informative message, check
```

Validation response form

Please find below a validation response form (VRF) that can be filled in and pasted into your CIF.

```
# start Validation Reply Form
_vrf_PLAT042_lcz002
;
PROBLEM: Calc. and Reported MoietyFormula Strings  Differ     Please Check
RESPONSE: ...
;
_vrf_PLAT214_lcz002
;
PROBLEM: Atom F020      (Anion/Solvent) ADP max/min Ratio        4.4 prolat
RESPONSE: ...
;
_vrf_PLAT221_lcz002
;
```

```
PROBLEM: Solv./Anion  Resd 2  F   Ueq(max)/Ueq(min) Range        4.2 Ratio
RESPONSE: ...
;
_vrf_PLAT234_lcz002
;
PROBLEM: Large Hirshfeld Difference F020     --C02D     .       0.16 Ang.
RESPONSE: ...
;
_vrf_PLAT250_lcz002
;
PROBLEM: Large U3/U1 Ratio for <U(i,j)> Tensor(Resd    2)        2.4 Note
RESPONSE: ...
;
_vrf_PLAT342_lcz002
;
PROBLEM: Low Bond Precision on  C-C Bonds ...............    0.00957 Ang.
RESPONSE: ...
;
_vrf_PLAT906_lcz002
;
PROBLEM: Large K Value in the Analysis of Variance ......      2.361 Check
RESPONSE: ...
;
_vrf_PLAT911_lcz002
;
PROBLEM: Missing FCF Refl Between Thmin & STh/L=    0.600         36 Report
RESPONSE: ...
;
_vrf_PLAT971_lcz002
;
PROBLEM: Check Calcd Resid. Dens.  1.27Ang From F01F           1.55 eA-3
RESPONSE: ...
;
# end Validation Reply Form
```

It is advisable to attempt to resolve as many as possible of the alerts in all categories. Often the minor alerts point to easily fixed oversights, errors and omissions in your CIF or refinement strategy, so attention to these fine details can be worthwhile. It is up to the individual to critically assess their own results and, if necessary, seek expert advice.

PLATON version of 17/09/2025; check.def file version of 17/09/2025

---

duplicate check

No duplication found

Datablock lcz002 - ellipsoid plot

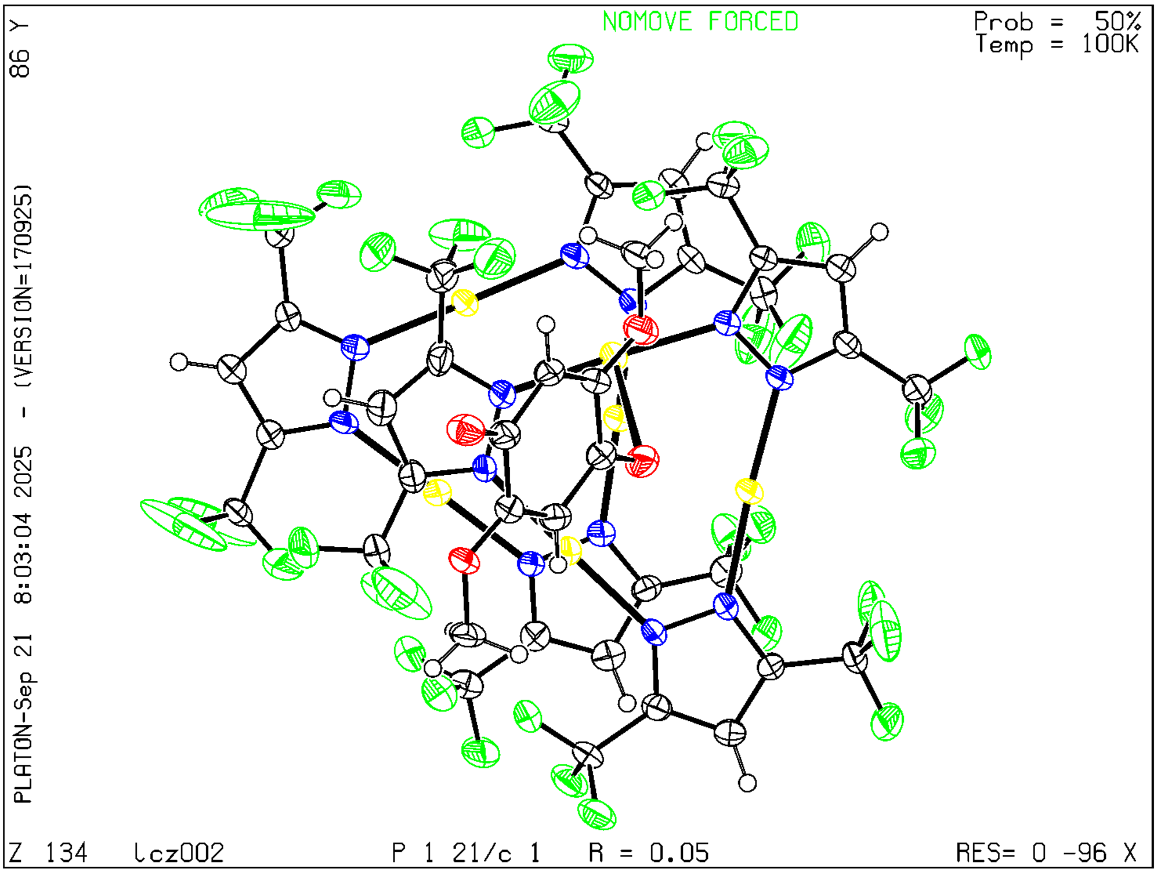

Supplement: Supplementary file 2 — Supporting File 2: anie72462‐sup‐0002‐DataFile.zip. [file ANIE-65-e1218503-s002.zip › supplementary_materials_crystallographic_data submit.zip!/cif_and_checkcif(1-114)/Ag3Pz3_2/Ag3Pz3_2_checkcif.pdf]
